# The impact of community-based home care service utilization on intergenerational family support: enhancement or attenuation?

**DOI:** 10.3389/fpubh.2026.1825288

**Published:** 2026-05-22

**Authors:** Meiling Du, Yanjun Chen, Liming Xiao, Shuai Liu

**Affiliations:** 1 School of Economics and Management, Shanxi Normal University, Taiyuan, China; 2 Business School, Beijing Normal University, Beijing, China

**Keywords:** community-based home care service utilization, family-based elder care, social elder care, intergenerational family support, intergenerational economic support, intergenerational instrumental support, intergenerational emotional support

## Abstract

**Introduction:**

As population aging intensifies, elder care has emerged as a central issue affecting socioeconomic sustainability, social harmony, and stability. The coordinated development of social care and family-based care has become pivotal to addressing the challenges of elder care. The rise of community-based home care services, while bridging gaps in family-provided care, has also exerted far-reaching effects on intergenerational family support. Therefore, examining the impact of community-based home care service utilization on intergenerational family support carries significant implications for addressing the elder care challenge.

**Methods:**

Drawing on data from the 2020 Chinese Longitudinal Aging Social Survey (CLASS) and given the ordinal nature of the dependent variables, this study employs an ordered Probit model, instrumental variable estimation (IV ordered Probit), and propensity score matching (PSM) to examine the effect of community-based home care service utilization on intergenerational family support.

**Results:**

Community-based home care service utilization significantly promotes intergenerational economic support, yet has a crowding-out effect on intergenerational instrumental and emotional support. Moderation analysis reveals that co-residence with adult children mitigates the adverse effects of community-based home care service utilization on intergenerational instrumental and emotional support. Heterogeneity analysis indicates that the negative effects on intergenerational instrumental and emotional support are primarily concentrated among urban households, with no statistically significant effect observed among rural households. In contrast, the positive effect on intergenerational economic support is more pronounced in urban households.

**Discussion:**

This study provides empirical evidence for understanding the relationship between community-based home care services and intergenerational family support. It delineates the differentiated effects and heterogeneous characteristics of community-based home care service utilization across distinct types of intergenerational support, yielding important policy implications for optimizing the elder care service system and fostering the coordinated development of social care and family-based care.

## 1 Introduction

The rise of community-based home care services, while bridging gaps in family-provided care, has also had far-reaching effects on intergenerational family support. As China’s socioeconomic development and demographic transition continue, population aging has become increasingly acute. By the end of 2025, the population aged 60 and above reached 323.38 million, accounting for 23.0% of the total population, while the population aged 65 and above reached 223.65 million, representing 15.9% of the total (47). China has entered a stage of moderate aging, and as household nuclearization and the prevalence of “empty- nest” households intensify, the scale of elder care demand continues to expand, and its composition becomes increasingly diverse (1). The traditional family-based care model faces mounting challenges stemming from shrinking household sizes and diminishing intergenerational support, rendering it increasingly inadequate to meet the growing complexity and multilayered nature of elder care needs, and thus necessitating broader societal engagement. In recent years, the Chinese government has introduced a series of policies that, while reaffirming the foundational role of family-based care, actively promote the development of community-supported home care. Against this backdrop, community-based home care services—by virtue of their proximity and accessibility—have emerged as a vital component of China’s elder care service system. Nevertheless, as professionalized social forces accelerate their involvement in family care provision, scholarly consensus has yet to be reached on whether intergenerational family support is strengthened by alleviating caregiving burdens or, conversely, weakened as elder care responsibilities shift outward from the family. A deeper investigation of this question not only contributes theoretically to advancing understanding of the “family–community” care nexus in the context of population aging, but also holds practical significance for informing the implementation of silver economy policies and refining a multi-tiered elder care service system.

Regarding the impact of community-based home care service utilization on intergenerational family support, existing research has generated extensive debate about crowding-out and crowding-in effects. However, a growing body of empirical evidence suggests that these two effects are not mutually exclusive; rather, they exhibit structural differentiation in opposing directions across distinct types of intergenerational support (2, 3). In the context of community-based home care services in particular, service provision may substitute for children’s instrumental care inputs while simultaneously reducing caregiving burdens in ways that facilitate emotional interaction and higher-quality companionship, thereby giving rise to a compound effect structure characterized by instrumental crowding out and emotional crowding in Sun et al. (4). This suggests that treating intergenerational support as a single, undifferentiated variable risks obscuring critical mechanistic heterogeneity in the context of formal service intervention. Intergenerational support theory, rooted in the sociological altruism model (5), is typically analyzed along three dimensions: economic, instrumental, and emotional support. With respect to economic support, existing studies have demonstrated that children’s financial transfers exert a significantly positive effect on older adults’ health and subjective well-being (6, 7); however, the extant literature has seldom directly examined, from a crowding-in perspective, how community-based service utilization affects intergenerational economic support through its influence on children’s labor time allocation. With respect to instrumental support, older adults’ demand for daily care increases steadily with age, and most exhibit a strong preference for family-based care arrangements (8); yet, given the substantial functional overlap between professional community services and children’s day-to-day caregiving, the crowding-out logic driven by substitution effects carries considerable empirical grounding along this dimension. With respect to emotional support, children’s psychosocial interaction with older adults is essential to the latter’s psychological well-being; in practice, however, emotional care is frequently overshadowed by material support (9, 10). More importantly, scholarly attention to the emotional support dimension remains relatively limited, with few studies subjecting it to systematic examination as an independent outcome variable (11).

Meanwhile, prevailing scholarship holds that the effective provision of community-based home care services should be grounded in the shared responsibilities of multiple stakeholders (12, 13). Service content has largely come to encompass such foundational domains as daily living assistance, healthcare, and psychological support (14, 15); nevertheless, a significant misalignment persists between supply and the actual needs of older adults (16, 17). Nevertheless, existing studies still have the following limitations. First, most scholars regard social care for older adults and family-based elder care as two independent systems, lacking an integrated analysis of their interactive mechanisms. Second, prior research predominantly focuses on macro-level policy effects, while insufficient attention has been paid to how the micro-level behavior of service utilization differentially affects the internal mechanisms of intergenerational support across various dimensions. Finally, there may be potential endogeneity between service utilization and intergenerational support, which has not been adequately addressed in most studies, further undermining the credibility of causal identification. Against this backdrop, this study draws on data from the 2020 Chinese Longitudinal Aging Social Survey (CLASS). It employs an Ordered Probit model in conjunction with instrumental variable estimation to disaggregate intergenerational support into three dimensions—economic, instrumental, and emotional—and systematically examines the effects of community-based home care service utilization on intergenerational family support and its underlying mechanisms, to provide empirical evidence and policy guidance for the development of an elder care service system suited to China’s national conditions.

Compared with existing studies, the marginal contributions of this research are evident in three respects. First, this study systematically uncovers the dimensional differentiation effects of community-based home care service utilization on intergenerational support by conducting a multidimensional decomposition of intergenerational support and separately evaluating the impact of service utilization across the economic, instrumental, and emotional dimensions. Second, by situating family-based and community-based care within a unified analytical framework and examining their interaction mechanisms from a micro-level perspective on service utilization behavior, this study addresses the limitations of prior research, characterized by the artificial separation of social care from family-based care and the disconnect between macro-level policy effects and household-level response mechanisms. Third, the application of instrumental variable estimation effectively addresses the endogeneity of service utilization; further examination of the moderating role of co-residence patterns and the heterogeneous effects of the urban–rural dual structure enhances the credibility of causal identification and deepens the understanding of differentiated pathways through which the “family-community integration” model of elder care may be realized.

The remainder of this paper is structured as follows. Section 2 presents the theoretical analysis and research hypotheses, drawing on relevant theories to examine how utilization of community-based home care services affects intergenerational family support and to derive the study’s hypotheses. Section 3 outlines the research design, detailing the data sources, model specification, and variable definitions. Section 4 reports the empirical results and analysis, including baseline regression, endogeneity issues, robustness tests, and further analysis. Section 5 discusses the effects of community-based home care service utilization on intergenerational family support, elaborating on the study’s arguments in light of the empirical findings and laying the groundwork for the conclusions. Section 6 presents the conclusions and policy recommendations, summarizing the key findings and proposing corresponding policy implications.

## 2 Theoretical analysis and research hypotheses

**Figure 1:**
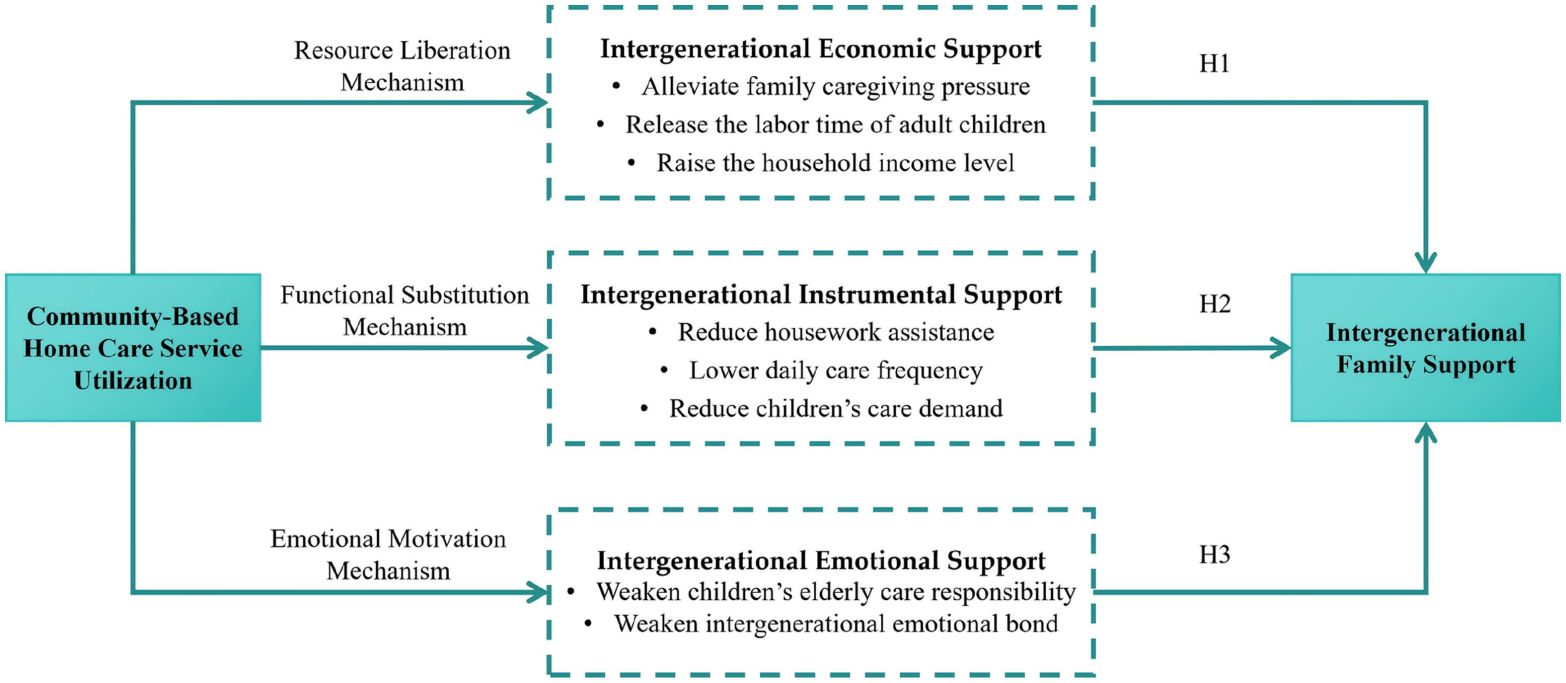
Theoretical analytical framework for the impact of community-based home care service utilization on intergenerational family support.

The intervention of community-based home care services fundamentally alters the allocation of time, income, and emotional resources within the family. Its impact on intergenerational family support is not unidirectional but rather constitutes a reallocation process shaped by the joint operation of multiple mechanisms. Existing studies have largely treated intergenerational support as a single, undifferentiated variable; however, a growing body of evidence suggests that distinct types of intergenerational support may exhibit divergent, even opposing, trajectories in response to service intervention. Against this backdrop, this study disaggregates intergenerational support into three dimensions—economic support, instrumental support, and emotional support—and, drawing on Becker's (18) household decision-making model and Grossman's (19) health demand model, together with social psychological theory, constructs an integrated analytical framework encompassing three mechanisms: resource release, functional substitution, and emotional motivation. Specifically, community-based home care services operate primarily through two economic rationality mechanisms and one social psychological mechanism: first, the resource release mechanism, whereby service provision substitutes for a portion of household caregiving inputs, thereby freeing up children’s time and influencing their labor supply and income levels; second, the functional substitution mechanism, whereby the functional overlap between community services and children’s caregiving leads households to engage in cost-efficiency trade-offs across different modes of care provision; and third, the emotional motivation mechanism, whereby service intervention alters individuals’ perceptions of family obligations and emotional needs, thereby influencing intergenerational interaction behaviors. These three mechanisms operate differently across distinct support dimensions and collectively constitute the theoretical foundation of this study. Therefore, this study constructs a theoretical analytical framework to elaborate the relationship between community-based home care service utilization and family intergenerational support, as shown in Figure 1.

### 2.1 Community-based home care service utilization and intergenerational economic support

Becker’s household decision-making model (18) conceptualizes the family as a unified unit of production and consumption, in which household members maximize family utility by allocating time and material resources. Central to this model is the insight that the distribution of resources within the household is subject to dual constraints of time and income, compelling family members to make trade-offs between labor market participation and family caregiving. Within this framework, the time children devote to parental care directly affects their labor supply and income-earning capacity, thereby further constraining the level of intergenerational economic support they can provide. In the absence of community-based home care services, children must dedicate substantial time to their parents’ daily care, which crowds out their market work hours, reduces their wage income, and, to some extent, diminishes their capacity to provide economic support to their parents. When community-based home care services are introduced and used, they assume some of the caregiving functions previously performed by children. This substitution process, operating through the resource release mechanism, effectively alleviates children’s time constraints and enables them to allocate more time to labor market participation. Holding wage rates constant, the increase in market working hours directly raises children’s wage income (20). Under the household budget constraint, higher income implies an expansion of disposable household resources; driven by altruistic motives or intergenerational exchange mechanisms, a substantial share of this incremental income is likely to be channeled into transfers from children to parents, thereby generating an income effect. Concurrently, the utilization of community-based home care services may itself entail certain financial costs, and as older adults attain a degree of material security through service provision, the perceived necessity of children’s economic transfers may correspondingly diminish, giving rise to a partial substitution effect. Nevertheless, compared with the aforementioned substitution mechanism, the income growth pathway generated by resource release is more direct and sustained; particularly within an institutional context in which the family continues to bear primary responsibility for elder care, increases in children’s income do not typically translate into a complete displacement of their filial economic support obligations (21). In sum, community-based home care service utilization alleviates household caregiving burdens. It releases family members’ labor time, thereby facilitating the rational reallocation of resources within the household, enhancing overall household welfare, and ultimately strengthening intergenerational economic support. Accordingly, the following research hypothesis is proposed.

*H1:* Community-based home care service utilization positively promotes intergenerational economic support by releasing children’s labor time and increasing household income.

### 2.2 Community-based home care service utilization and intergenerational instrumental support

Grossman’s health demand model (19) conceptualizes health as a form of human capital that households produce and maintain through investment in time and market goods. Extending this model to the elder care context, older adults’ capacity for independent living may be understood as a form of “welfare capital” that requires continuous production, with its generation depending on multiple inputs—encompassing both intergenerational instrumental support from within the family and professional care services provided by the community. From the perspective of the technical characteristics of the production function, community-based home care services and children’s intergenerational support exhibit a high degree of functional overlap along the instrumental dimension, as both primarily aim to meet older adults’ basic needs in areas such as daily living and household assistance. Within a household utility maximization framework, different modes of care provision are clearly substitutable, and rational households will select the optimal input combination subject to cost and efficiency constraints. Given that community professional services typically benefit from economies of scale and a specialized division of labor, their output efficiency per unit cost tends to exceed that of children’s non-professional, fragmented caregiving. Consequently, households are inclined to substitute community-based home care services for intergenerational instrumental support—that is, to reduce children’s caregiving inputs and rely instead on community services (22). In other words, as the frequency and coverage of community-based home care service utilization increase, the probability and frequency with which children provide instrumental support, such as household help and daily care, decline significantly. Older adults themselves may also proactively reduce their reliance on children’s care as they become accustomed to the convenience and professionalism of formal services, giving rise to “demand-side substitution” and resulting in a net reduction in intergenerational instrumental support at the household level (23). In sum, as the accessibility and professionalism of community-based home care services continue to improve, older adults gradually become more reliant on these services and reduce their demand for instrumental care from children. Accordingly, the following research hypothesis is proposed.

*H2:* Community-based home care service utilization reduces intergenerational instrumental support through a substitution effect, attributable to its functional overlap with children’s caregiving.

### 2.3 Community-based home care service utilization and intergenerational emotional support

The impact of community-based home care service utilization on intergenerational emotional support is examined through two theoretical lenses: the responsibility dilution hypothesis and socioemotional selectivity theory. On the one hand, the responsibility dilution hypothesis holds that when a task or obligation can be shared among multiple actors, each actor’s perceived personal responsibility diminishes. In the elder care context, once community-based care services intervene, caregiving responsibility expands from the family as a sole provider to a pluralistic “family–community” arrangement. Children may subconsciously perceive that their parents are already receiving care and companionship from service providers and, consequently, feel justified in moderating their own emotional investment (24). This dilution of responsibility, however, does not necessarily reflect indifference on the part of children; rather, it represents an unconscious psychological adjustment. On the other hand, drawing on socioemotional selectivity theory (25, 26), when community-based home care services can provide stable, reliable emotional comfort, older adults’ psychological need for emotional dependence on their children may diminish. They may partially redirect their emotional expectations toward service providers, thereby reducing how often they actively seek emotional support from their children. In this process, the emotional demand signals that children receive weaken, further attenuating their motivation to invest emotionally. In sum, the proliferation of community-based home care services may, through the responsibility dilution mechanism, erode children’s sense of filial obligation and lead them to redirect greater energy toward personal pursuits; the socioemotional selectivity mechanism further reinforces older adults’ reliance on external care services, thereby weakening their emotional bonds with children. The joint operation of these two mechanisms may result in a net reduction in intergenerational emotional support following service utilization. It should be noted that such a reduction does not necessarily signify a deterioration in intergenerational relations. Still, it may instead reflect a reconfiguration of caregiving responsibilities and a diversification of channels for meeting emotional needs. Accordingly, the following research hypothesis is proposed.

*H3:* Community-based home care service utilization reduces intergenerational emotional support through the mechanisms of responsibility dilution and socioemotional selectivity.

## 3 Research design

### 3.1 Data sources

**Figure 2:**
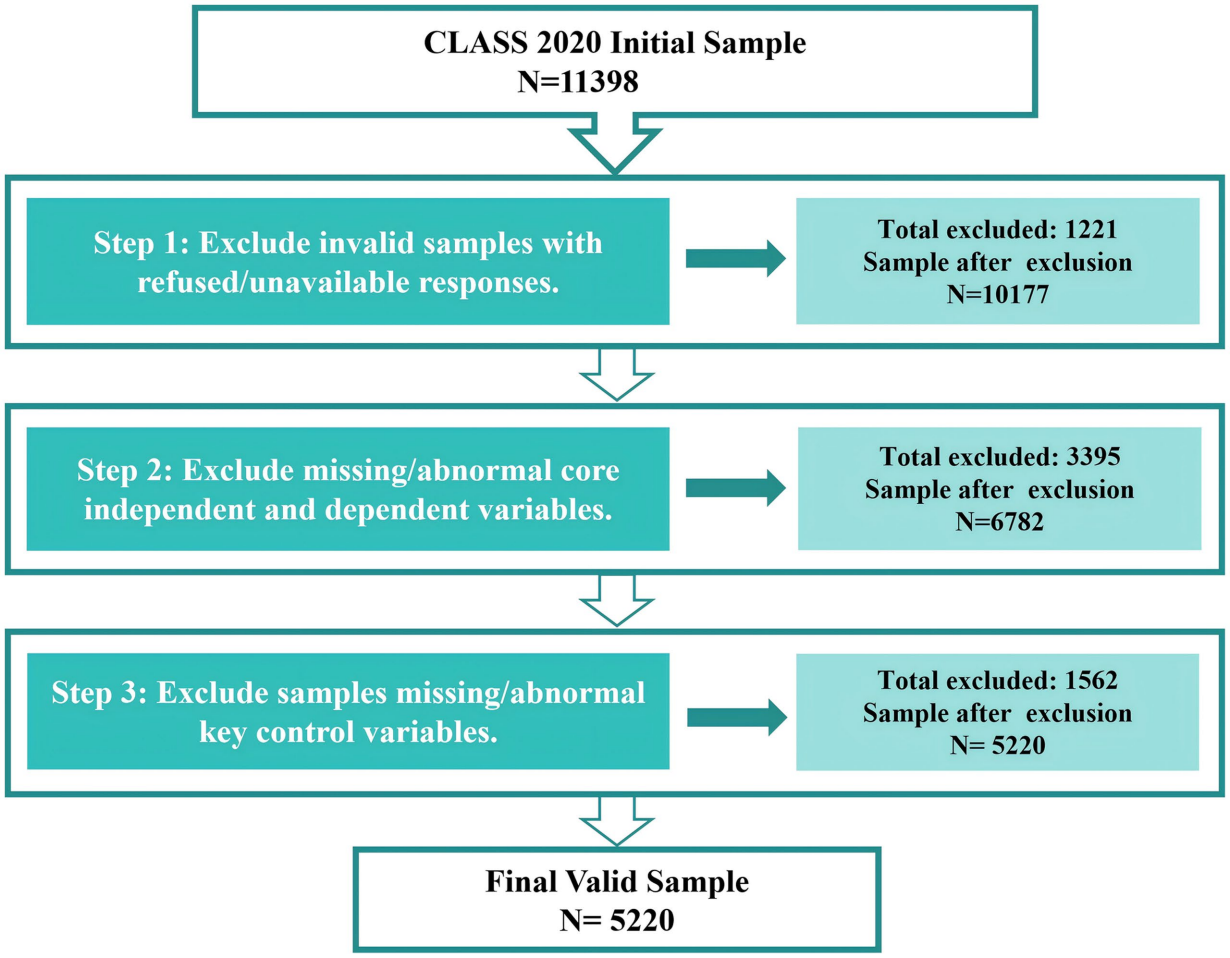
Schematic diagram of the sequential screening procedure for the analytic sample.

The data for this study are drawn from the 2020 China Longitudinal Aging Social Survey (CLASS). CLASS is a nationally representative longitudinal survey of the aging population, employing a stratified, multi-stage probability sampling design that covers 28 provinces, autonomous regions, and municipalities. The survey encompasses data on the socioeconomic conditions, health status, family circumstances, and social support of adults aged 60 and above, providing a reliable data foundation for the present study. To ensure sample validity and analytical reliability, the raw data were subjected to a sequential screening procedure as follows (see Figure 2). In the first step, an initial sample screening was conducted. The raw sample of the 2020 CLASS dataset comprised 11,398 observations; after excluding invalid samples with refused or unavailable responses, 10,177 observations remained. In the second step, observations with missing values on core variables were removed. The core dependent variables are intergenerational economic, instrumental, and emotional support, while the core independent variable is community-level utilization of elder care services. After excluding observations with missing or anomalous values on any of these four variables, 6,782 observations remained. In the third step, observations with missing values on key control variables were addressed. Key control variables include gender, educational attainment, pension insurance coverage, self-rated health, activities of daily living (ADL), number of chronic conditions, marital status, number of children, and number of co-residing household members. Observations with a substantial number of missing values on control variables were removed. In contrast, those with relatively few missing values were imputed using multiple imputation. Finally, a final analytic sample of 5,220 observations was obtained for the subsequent empirical analysis.

### 3.2 Variable description

#### 3.2.1 Independent variables

Community-Based Home Care Service Utilization. The core measure for this variable is derived from the survey question: “Over the past year, have you used any elder care services provided by the community?” Older adults who reported having utilized such services were assigned a value of 1, while those who had not were assigned a value of 0.

#### 3.2.2 Dependent variables

Intergenerational Support. Drawing on the classification of intergenerational support proposed by Wang et al. (27) and integrating Maslow’s hierarchy of needs, this study disaggregates intergenerational support into three dimensions: economic, instrumental, and emotional. Specifically, intergenerational economic support is measured by the survey question: “Over the past year, how much cash or in-kind support did your children provide to you in total?” Intergenerational instrumental support is captured by the survey question: “Over the past year, how frequently did your children help with household chores or assist with your daily care in total?” Intergenerational emotional support is assessed by the survey question: “Over the past year, how frequently did you communicate with your children in total, through means such as in-person visits, telephone calls, or the internet?” It should be noted that the level of intergenerational support recorded here reflects the aggregate support provided by all children. For older adults with multiple children, the level of support each child provides is first calculated, then either summed or averaged across all children.

#### 3.2.3 Control variables

Beyond the utilization of community-based home care services, a wide range of factors may influence intergenerational family support. To minimize estimation bias arising from omitted variable bias, this study draws on prior research (28, 29) and selects a set of control variables at both the individual and household levels. At the individual level, the following variables are included: gender, level of education, presence of pension insurance, self-rated health, activities of daily living (ADL), and number of chronic conditions, to capture both the subjective perceptions and objective circumstances that shape older adults’ demand for elder care services. At the household level, marital status, number of children, and number of household members are included to account for the potential influence of intra-household human resource allocation and structural characteristics on elder care decisions. Variable definitions and descriptive statistics are presented in Table 1.

**Table 1:** Variable definitions and descriptive statistics.

Variable	Variable name	Abbreviations	Definition	Mean	S.D.	*N*
Independent variable	Intergenerational economic support	IECS	Total value of material or cash support provided by children over the past year: Less than 1,000 yuan = 1; 1,000 ~ 2,000 yuan = 2; 2,000 yuan and above = 3	1.990	0.865	5,220
	Intergenerational instrumental support	IINS	Frequency of children providing domestic assistance and personal care over the past year: Rarely = 1; Occasionally = 2; Frequently = 3	2.215	0.829	5,220
	Intergenerational emotional support	IEMS	Frequency of contact with children over the past year: Rarely = 1; Occasionally = 2; Frequently = 3	2.396	0.529	5,220
Dependent variable	Community-based home care service utilization	CBHCS	Over the past year, have you utilized any community-based elder care services: Yes = 1; No = 0	0.108	0.310	5,220
Instrumental variable	Community-based home care provision	CBHCP	Does the community provide social care for older adults: Yes = 1; No = 0	0.486	0.500	5,220
Control variables	Gender	Gender	Male = 1; Female = 0	0.504	0.500	5,220
	Level of education	Edu	No education = 1; Primary or literacy education = 2; Education beyond primary level = 3	2.165	0.758	5,220
	Presence of pension insurance	Insurance	Yes = 1; No = 0	0.881	0.324	5,220
	Self-rated health	Health	Healthy = 1; Fairly well = 2; Unwell = 3	2.268	0.725	5,220
	Activities of Daily Living	ADL	Fully independent = 0; Requires some assistance = 1; Unable to self-care = 2	0.791	0.407	5,220
	Number of chronic conditions	Chronic	0 item = 1; 1 item = 2; 2 items = 3; 3 items and above = 4	2.731	1.232	5,220
	Marital status	Marital	No spouse including widowed = 0; With spouse = 1	0.755	0.430	5,220
	Number of children	Children	0 item = 1; 1 item = 2; 2 items = 3; 3 items = 4; 4 items and above = 5	3.316	1.075	5,220
	Number of household members	Household	0 item = 1; 1 item = 2; 2 items = 3; 3 items = 4; 4 items and above = 5	3.536	0.962	5,220

### 3.3 Model specification

#### 3.3.1 Ordered probit model

Given that the dependent variable “intergenerational family support” is a discrete ordinal variable, direct application of OLS regression would yield biased parameter estimates. Accordingly, this study employs the Ordered Probit model for baseline estimation, thereby circumventing the parameter-estimation bias and heteroskedasticity problems that arise from treating ordinal variables as continuous. To examine the effects of community-based home care service utilization on intergenerational economic support, intergenerational instrumental support, and intergenerational emotional support, the following model is specified:

$$\text{Suppor}t_{\mathrm{ij}}^{*}=\alpha +\mathrm{\beta Us}e_{i}+\gamma X_{i}+\varepsilon_{i}$$ (1)

$$\text{Suppor}t_{\mathrm{ij}}=\{\begin{array}{c} \;1,\;\;\;\text{Suppor}t_{\mathrm{ij}}^{*}\leq \mu_{1}\\ 2,\mu_{1}<\text{Suppor}t_{\mathrm{ij}}^{*}\leq \mu_{2}\\ 3,\mu_{2}<\text{Suppor}t_{\mathrm{ij}}^{*}\leq \mu_{3} \end{array}$$ (2)

where $\text{Suppor}t_{\mathrm{ij}}^{*}$ denotes the latent variable for individual i on the j−th dimension of intergenerational support (economic support, instrumental support, and emotional support); $\text{Suppor}t_{\mathrm{ij}}$ is the corresponding observed dependent variable; $\mathrm{Us}e_{i}$ indicates whether individual i utilizes community-based home care services; $X_{i}$ is a vector of control variables; β , γ , and δ are estimated coefficients; $\varepsilon_{i}$ is a random error term following a standard normal distribution; and $\mu_{1}<\mu_{2}<\mu_{3}$ denotes the threshold parameter.

#### 3.3.2 IV ordered probit model

The core dependent variables in this study (intergenerational economic support, intergenerational instrumental support, and intergenerational emotional support) are all ordinal polytomous variables. In contrast, the core independent variable (community-based home care service utilization) is a binary choice variable. A potential bidirectional causal relationship between these variables may give rise to endogeneity in the model specification described above. Specifically, whether older adults utilize community-based home care services may be influenced by unobservable factors such as health status, economic conditions, and children’s willingness to provide support—factors that may simultaneously affect both the level and composition of intergenerational support, thereby introducing bias into the estimates yielded by the conventional Ordered Probit model. To accurately identify the causal effect of community-based home care service utilization on intergenerational support, this study employs a combined instrumental-variables and conditional mixed process (CMP) approach to address endogeneity in the baseline regression (30). This study selects “whether the community provides elder care services” as the instrumental variable for “whether the individual utilizes such services.” This variable is measured by the CLASS survey question: “Does the community in which you reside provide elder care services?” with communities that provide such services coded as 1 and those that do not coded as 0. This variable reflects the coverage of elder care services at the community level. On the one hand, the availability of community services directly influences the likelihood that older adults will use them; on the other hand, community-level service provision decisions are relatively independent of individual household intergenerational support behavior. The proposed instrumental variable, therefore, satisfies both the relevance and exogeneity conditions.

## 4 Empirical results and analysis

### 4.1 Baseline regression analysis

Based on Equations 1, 2, we obtain the regression estimation results of the ordered probit model (see Table 2). With respect to intergenerational economic support, the results in column (1) of Table 2 show that the coefficient on community-based home care service utilization is significantly positive at the 1% level. This indicates that, holding other factors constant, older adults who use community-based home care services are significantly more likely to receive higher levels of intergenerational economic support than their non-users. This finding confirms a significant positive effect of community-based home care service utilization on intergenerational economic support, consistent with Han et al. (31) and thereby validating Hypothesis 1. A plausible explanation is twofold: on the one hand, service utilization releases children’s labor time and increases household income, thereby enhancing children’s capacity to provide economic support to their parents; on the other hand, service utilization alleviates household caregiving burdens, enabling families to channel more resources into productive activities and generating a virtuous cycle of strengthened intergenerational economic support.

**Table 2:** Baseline regression results for the effect of community-based home care service utilization on intergenerational family support.

Variable	Intergenerational economic support	Intergenerational instrumental support	Intergenerational emotional support
	(1)	(2)	(3)
CBHCS	0.528*** (0.061)	−0.094*** (0.036)	−0.087** (0.042)
Gender	−0.148*** (0.053)	−0.210*** (0.060)	−0.402*** (0.083)
Edu	0.378*** (0.037)	0.068*** (0.024)	0.226*** (0.057)
Insurance	−0.112 (0.082)	−0.034 (0.092)	0.548*** (0.108)
Health	0.272*** (0.044)	0.182*** (0.046)	−0.215*** (0.058)
ADL	0.156*** (0.039)	0.103** (0.049)	−0.176** (0.069)
chronic	0.074*** (0.024)	0.062** (0.028)	−0.081* (0.042)
Marital	0.135** (0.063)	−0.642*** (0.078)	−0.235** (0.099)
Children	0.034 (0.026)	−0.258*** (0.030)	−0.350*** (0.039)
Household	0.106*** (0.027)	0.905*** (0.038)	0.627*** (0.051)
Constant	−1.623*** (0.192)	−1.422*** (0.223)	0.758*** (0.288)
*N*	5,220	5,220	5,220

*, **, and *** denote statistical significance at the 10, 5, and 1% levels, respectively; robust standard errors are reported in parentheses.

Regarding intergenerational instrumental support, the results in column (2) of Table 2 show that the coefficient on community-based home care service utilization is −0.094, indicating that utilization of community-based elder care services is associated with a significant 9.40 percentage point reduction in the probability of receiving higher levels of intergenerational instrumental support. This finding demonstrates that community-based home care service utilization exerts a significant crowding-out effect on intergenerational instrumental support, consistent with the conclusions of Wang et al. (32), thereby validating Hypothesis 2. A plausible explanation lies in the substantial functional overlap between community-based home care services and children’s instrumental caregiving: when professional community services can meet older adults’ daily living needs, children correspondingly reduce their direct caregiving. This crowding-out effect lends support to the “functional substitution” hypothesis (33).

Regarding intergenerational emotional support, the results in column (3) of Table 2 show that the coefficient on community-based home care service utilization is −0.087, indicating that utilization of community-based elder care services is associated with a significant 8.70 percentage point reduction in the probability of receiving higher levels of intergenerational emotional support. This finding confirms that community-based home care service utilization likewise exerts a crowding-out effect on intergenerational emotional support, thereby validating Hypothesis 3. The underlying mechanism may be attributed to “responsibility dilution.” Once community services intervene, service utilization attenuates children’s sense of urgency about their caregiving obligations to their parents, thereby reducing the frequency of emotional contact. Concurrently, older adults may experience a diminished psychological need for emotional support from children due to the emotional comfort provided by service personnel (34). As the control variables are not the primary focus of this study, a detailed discussion of their effects is omitted here for brevity.

### 4.2 Endogeneity issues

Although the baseline regression results indicate that community-based home care service utilization has a significant effect on intergenerational family support, potential reverse causality and omitted variable bias may introduce endogeneity in the core independent variable, rendering the baseline regression coefficient estimates biased and inconsistent. Accordingly, this study employs instrumental-variables estimation using the IV Ordered Probit model to address potential endogeneity. Column (1) of Table 3 reports the first-stage regression results and endogeneity test statistics. The first-stage F-statistic is 46.28, exceeding the conventional threshold of 10, indicating the absence of a weak instrument problem. The estimated coefficient of community-level elder care service provision on service utilization is 0.387 and is statistically significant at the 1% level, satisfying the relevance condition for a valid instrument. Columns (2) through (4) of Table 3 report the IV Ordered Probit estimation results. The coefficient atanhrho is statistically significant at the 1% level, thereby rejecting the null hypothesis that community-based home care service utilization is exogenous and confirming the appropriateness of the selected instrumental variable. The estimation results reveal that the coefficients on community-based home care service utilization with respect to intergenerational economic support, instrumental support, and emotional support are each statistically significant at least at the 5% level and are somewhat larger than the baseline regression estimates. These findings demonstrate that, after accounting for endogeneity, community-based home care service utilization continues to exert significant and differentiated effects on intergenerational family support—effects that are in fact more pronounced—further corroborating the research hypotheses advanced earlier in this study.

**Table 3:** Endogeneity test and IV ordered probit estimation results.

Variable	First stage	Second stage		
	CBHCS	Intergenerational economic support	Intergenerational instrumental support	Intergenerational emotional support
	(1)	(2)	(3)	(4)
CBHCP	0.387*** (0.032)			
CBHCS		0.567*** (0.032)	−0.104** (0.043)	−0.101* (0.057)
Control variables	Yes	Yes	Yes	Yes
Wald $\chi^{2}$		186.64***	221.90***	144.11***
First-stage F-statistic	46.28			
atanhrho		0.126***	−0.179***	−0.201***
*N*	5,220	5,220	5,220	5,220

*, **, and *** denote statistical significance at the 10, 5, and 1% levels, respectively; robust standard errors are reported in parentheses.

### 4.3 Robustness tests

#### 4.3.1 Propensity score matching estimation

Although the instrumental variable approach addresses endogeneity to a considerable extent, sample selection bias may still be present in the context of community-based home care service utilization. This study, therefore, further employs propensity score matching (PSM) as a robustness check, estimating the average treatment effect of community-based home care service utilization by constructing a counterfactual framework to mitigate the confounding influence of self-selection bias on the findings (35). Specifically, community-based home care service utilization is designated as the treatment variable, while intergenerational economic support, intergenerational instrumental support, and intergenerational emotional support serve as outcome variables; the original set of control variables is incorporated into the covariate pool. To ensure the reliability of the matching results, a covariate balance test is first conducted. Table 4 presents the balance test results for each covariate before and after matching. The results indicate that, following matching, the absolute standardized bias for all covariates is contained within 10%, the mean differences in control variables between the treatment and control groups are negligible, and the *p*-values for the matched sample are substantially higher than those before matching, collectively confirming that the matching quality is satisfactory and that the balance test is passed.

**Table 4:** Covariate balance test results before and after propensity score matching.

Variable	Matching status	Mean		Standardized bias (%)	Bias reduction (%)	T-test	
		Treatment group	Control group			t-value	*p*-value
Gender	Before matching	0.452	0.510	−11.6	86.2	−2.58	0.010***
	after matching	0.432	0.460	−1.6		−0.24	0.808
Edu	Before matching	2.354	2.142	28.0	87.9	6.18	0.000***
	After Matching	2.285	2.328	3.4		0.53	0.594
Insurance	Before matching	0.925	0.876	16.5	89.1	3.37	0.001***
	After matching	0.907	0.920	1.8		0.31	0.756
Health	Before matching	2.412	2.251	22.2	83.8	4.91	0.000***
	After matching	2.426	2.386	3.6		0.55	0.580
ADL	Before matching	0.892	0.779	27.8	86.0	5.91	0.000***
	After matching	0.814	0.876	3.9		0.62	0.535
chronic	Before Matching	2.968	2.702	21.5	79.5	4.68	0.000***
	After matching	2.907	2.914	4.4		0.69	0.490
Marital	Before matching	0.785	0.751	8.0	67.5	1.68	0.094
	After matching	0.742	0.774	2.6		0.41	0.682
Children	Before matching	3.258	3.325	−6.2	54.8	−1.33	0.183
	After matching	3.286	3.287	−2.8		−0.43	0.665
Household	Before matching	3.487	3.542	−5.7	57.9	−1.21	0.226
	After matching	3.243	3.510	−2.4		−0.37	0.709

*, **, and *** indicate significance levels of 10%, 5%, and 1%, respectively.

Building on the foregoing matching and diagnostic results, this study further employs nearest-neighbor matching and radius matching to estimate the average treatment effect (ATE) of community-based home care service utilization on intergenerational family support (see Table 5). The results presented in Table 5 indicate that, under both matching methods, the average treatment effects of community-based home care service utilization on intergenerational economic support, intergenerational instrumental support, and intergenerational emotional support are each statistically significant at least at the 5% level, findings that are broadly consistent with the preceding regression results and further corroborate the robustness of the study’s conclusions.

**Table 5:** Average treatment effects from propensity score matching.

Dependent variable	Matching method	Average treatment effect	Standard error	t-value
Intergenerational economic support	Nearest-neighbor matching	0.478***	0.030	15.93
	Radius matching	0.461***	0.028	16.46
Intergenerational instrumental support	Nearest-neighbor matching	−0.086***	0.031	−2.77
	Radius matching	−0.079***	0.029	−2.72
Intergenerational emotional support	Nearest-neighbor matching	−0.065**	0.027	−2.41
	Radius matching	−0.062***	0.023	−2.70

*, **, and *** indicate significance levels of 10%, 5%, and 1%, respectively.

#### 4.3.2 Additional robustness tests

To further validate the robustness of the baseline regression results, this study conducts two supplementary checks. The first involves replacing the estimation model: an Ordered Logit model is employed to re-estimate the baseline specifications, with results reported in columns (1) through (3) of Table 6. The second involves replacing the measure of intergenerational emotional support: the alternative indicator “degree of emotional closeness with children” is adopted, with estimation again performed using the Ordered Probit model. In the baseline regression, intergenerational emotional support is measured by “frequency of contact or in-person visits with children,” which captures the frequency dimension of intergenerational interaction. However, the core conceptual content of emotional support lies in the quality of emotional bonds; given that contact frequency alone is insufficient to capture the construct of emotional support fully, this study further adopts the CLASS survey item “Considering all aspects, do you feel emotionally close to this child?” as an alternative indicator for supplementary validation. This variable reflects older adults’ subjective assessment of emotional intimacy with their children and more directly captures the qualitative dimension of intergenerational emotional support, thus serving as an effective complement to the contact frequency measure. In terms of variable coding, this variable is recoded as: not close = 0, somewhat close = 1, and close = 2. The estimation results are reported in column (4) of Table 6. The results obtained under both approaches are consistent with the baseline regression findings reported in Table 2, further confirming the robustness of the study’s conclusions.

**Table 6:** Results of additional robustness tests.

Variable	Intergenerational economic support	Intergenerational instrumental support	Intergenerational emotional support	Intergenerational emotional support (Closeness)
	(1)	(2)	(3)	(4)
CBHCS	0.954*** (0.053)	−0.158*** (0.061)	−0.312*** (0.117)	−0.187** (0.089)
Control variables	Yes	Yes	Yes	Yes
*N*	5,220	5,220	5,220	5,220

*, **, and *** indicate significance levels of 10%, 5%, and 1%, respectively; robust standard errors are reported in parentheses.

### 4.4 Further analysis

#### 4.4.1 Moderation analysis of co-residence patterns

For the potential supportive effects of community-based home care service utilization to translate into actual levels of intergenerational support, older adults’ residential arrangements are a critical boundary condition, with co-residence with adult children as the key determinant. While existing studies have confirmed the direct effect of residential arrangements on intergenerational support (36), few have systematically examined the moderating mechanisms through which co-residence operates in the process by which community-based home care service utilization is converted into intergenerational support outcomes. To test this moderating effect, this study introduces an interaction term, “community-based home care service utilization × residential arrangement,” into the baseline regression model. Following Liu and Kou (37), residential arrangement is defined based on the survey question: “Who shares the same household and meals with you?” If the response includes adult children, the older adult is classified as “co-residing with children” and assigned a value of 1; otherwise, a value of 0 is assigned. The results of the moderation effect test for residential arrangement are presented in Table 7.

**Table 7:** Moderating effect test results of co-residence patterns.

Variable	Intergenerational economic support	Intergenerational instrumental support	Intergenerational emotional support
	(1)	(2)	(3)
CBHCS	0.548*** (0.066)	−0.101** (0.038)	−0.089** (0.043)
Residential arrangement	−0.146** (0.072)	−0.262*** (0.078)	−0.148 (0.104)
CBHCS × Residential arrangement	0.122 (0.093)	0.354*** (0.125)	0.261* (0.150)
Constant	−1.734*** (0.212)	−1.323*** (0.226)	0.821*** (0.293)
Control variables	Yes	Yes	Yes
*N*	5,220	5,220	5,220

*, **, and *** indicate significance levels of 10%, 5%, and 1%, respectively; robust standard errors are reported in parentheses.

The regression results in Table 7 indicate that residential arrangement significantly moderates the relationship between community-based home care service utilization and intergenerational support, with the moderating effect varying across dimensions of intergenerational support. First, the moderating effect of residential arrangement on intergenerational economic support is not statistically significant. The coefficient on the interaction term between community-based home care service utilization and residential arrangement (0.122) is insignificant, indicating that co-residence with adult children does not alter the positive effect of service utilization on intergenerational economic support. This may be attributable to the proliferation of digital technologies, which has transcended geographical constraints and enabled children to conduct financial transfers conveniently even when living apart, thereby rendering the moderating role of residential arrangement ineffective with respect to economic support (38). Second, residential arrangement exerts a significant positive moderating effect on intergenerational instrumental support. The coefficient on the interaction term between community-based home care service utilization and residential arrangement (0.354) is positive and significant at the 1% level, indicating that co-residence with adult children significantly attenuates the crowding-out effect of service utilization on intergenerational instrumental support. In other words, when children reside with their parents, they continue to receive a relatively high frequency of instrumental caregiving inputs, even when parents use community-based home care services. This finding is consistent with Confucian cultural traditions and family altruistic motivations: co-residence facilitates closer daily interaction through spatial proximity, enabling children to more readily perceive their parents’ actual needs and thereby proactively sustain caregiving behaviors that offset the substitution effect of service utilization. Third, residential arrangement exerts a significant positive moderating effect on intergenerational emotional support. The coefficient on the interaction term between community-based home care service utilization and residential arrangement (0.261) is positive and significant at the 10% level, indicating that co-residence with adult children mitigates the adverse effect of service utilization on intergenerational emotional support. Drawing on Becker’s household utility theory (18), co-residence facilitates more effective integration of family resources, and face-to-face daily communication provides a natural platform for emotional bonding, thereby attenuating the “responsibility dilution” or emotional estrangement that may otherwise result from service utilization.

#### 4.4.2 Heterogeneity analysis under the urban–rural dual structure

China’s urban–rural economic development remains characterized by persistent imbalance. On the one hand, the elder care service system in urban areas is comparatively well-developed, with higher levels of service accessibility and professionalization; on the other hand, rural areas are constrained by lower levels of economic development and limited public service provision capacity, resulting in substantially lower rates of community-based elder care service coverage and utilization. Consequently, under China’s urban–rural dual economic structure, the effect of community-based home care service utilization on intergenerational family support may exhibit heterogeneity between urban and rural areas. To test this hypothesis, the full sample is divided into urban and rural subsamples, and separate regression estimations are conducted for each. The results are reported in Table 8.

**Table 8:** Heterogeneity regression results under the urban–rural dual structure.

Variable	Urban			Rural		
	Intergenerational economic support	Intergenerational instrumental support	Intergenerational emotional support	Intergenerational economic support	Intergenerational instrumental support	Intergenerational emotional support
	(1)	(2)	(3)	(4)	(5)	(6)
CBHCS	0.635*** (0.084)	−0.124*** (0.046)	−0.116*** (0.038)	0.404*** (0.078)	−0.087 (0.091)	−0.098 (0.111)
Constant	−2.375*** (0.340)	−1.729*** (0.385)	0.984* (0.509)	−2.107*** (0.282)	−1.083*** (0.284)	0.747** (0.362)
Control variables	Yes	Yes	Yes	Yes	Yes	Yes
*N*	2,663	2,663	2,663	2,557	2,557	2,557

*, **, and *** indicate significance levels of 10%, 5%, and 1%, respectively; robust standard errors are reported in parentheses.

The regression results in Table 8 reveal significant urban–rural heterogeneity in the effects of community-based home care service utilization on intergenerational family support. First, the effect of community-based home care service utilization on intergenerational economic support is significantly positive in both urban and rural households. Still, this promoting effect is more pronounced among urban households. This differential may stem from the more mature elder care service market in urban areas, where higher levels of service accessibility and professionalization enable service utilization to more effectively release children’s labor time and translate it into higher income and enhanced capacity for economic support; in rural areas, by contrast, the high proportion of informal employment limits the income-enhancing effect of time released through labor market reallocation (39). Second, the negative effects of community-based home care service utilization on both intergenerational instrumental and emotional support are statistically significant among urban households but not among rural households. This pattern may be attributable to two factors. On the one hand, overall insufficient supply and limited coverage of community-based home care services in rural areas result in lower rates of service utilization, such that service use has yet to reach the threshold at which it exerts a significant impact on intergenerational instrumental and emotional support. On the other hand, rural areas traditionally maintain relatively close community and neighborhood ties, and these informal social support networks partially buffer the impact of professional services on household caregiving functions and also moderate reductions in the frequency of emotional interactions with children (40). It is also worth noting that the stronger substitution effect of community-based home care service utilization on emotional support in urban households is likely related to the measurement approach adopted for emotional support. In the baseline regression of this study, emotional support is primarily measured by frequency of contact; given the relatively higher penetration and utilization rates of digital communication in urban households, where intergenerational relationships are more commonly maintained through telephone and digital means, the data may exhibit a more pronounced crowding-out effect in the urban context.

## 5 Discussion

Against the dual backdrop of deepening population aging and the nuclearization of family structures, community-based home care services, as a prominent form of social elder care, have attracted growing scholarly and policy attention regarding their mechanisms of influence on intergenerational family support. Unlike existing studies that predominantly focus on the current state of elder care service utilization and its determinants (41), or on the effects of elder care services on self-rated health (42, 43), life satisfaction (44), and family caregiving (45), this study adopts a multidimensional perspective on intergenerational support. It constructs a tripartite analytical framework encompassing resource release, functional substitution, and emotional motivation mechanisms, systematically examining the differentiated effects of community-based home care service utilization on three types of intergenerational support: economic, instrumental, and emotional. The findings not only illuminate the structural differentiation of intergenerational support under formal service intervention but also offer a novel theoretical perspective on the dynamic evolution of the “family–community” elder care relationship.

First, community-based home care service utilization has a significant positive effect on intergenerational economic support but exhibits crowding-out effects on instrumental and emotional support. This finding is highly consistent with the “structural differentiation” perspective advanced by Zhang and Yu (46), further corroborating that service intervention does not unidirectionally undermine family functions but rather produces heterogeneous outcomes contingent on the type of support in question. Second, co-residence with adult children significantly mitigates the negative crowding-out effects of community-based home care service utilization on instrumental support and emotional support. The significance of this moderating effect is particularly pronounced along the instrumental support dimension, reflecting the critical role of spatial proximity in sustaining family caregiving functions. Third, the crowding-out effects of community-based home care service utilization on instrumental support and emotional support are statistically significant only among urban households, with no significant effect observed among rural households. This finding aligns with the conclusions of Gonfa et al. (40): under low service coverage, socialized care provision is insufficient to induce systematic substitution for informal caregiving; consequently, rural elder care policy design should prioritize the preservation and activation of informal social support networks rather than simply replicating urban models. It is worth emphasizing that although the use of community-based home care services can crowd out instrumental and emotional support, this does not imply that older adults’ use of community-based elder care services should be restricted. On the contrary, the crowding-out effect itself reflects an optimization of the allocation of elder care resources and an improvement in caregiving efficiency. The critical challenge lies in preventing the attenuation of intergenerational relationships as service interventions proceed, through institutional design and service innovation. As the analysis in this study suggests, judicious guidance of residential arrangements, the “intergenerationally friendly” design of service content, and the emotional supplementation afforded by digital technologies all constitute effective pathways to mitigate crowding-out effects and foster the integration of family- and community-based elder care.

Although this study advances the understanding of the relationship between community-based home care services and intergenerational support, several limitations warrant acknowledgment. First, while the Chinese Longitudinal Aging Social Survey (CLASS) data are nationally representative, this study relies on cross-sectional data from 2020, which precludes tracking the dynamic changes within the same households before and after service utilization. Future research could leverage multi-wave CLASS panel data and employ methods such as PSM-DID to identify the dynamic effects of policy implementation over time more robustly. Second, the measurement of intergenerational support remains primarily based on frequency and monetary amounts, and thus fails to capture its qualitative dimensions adequately. For instance, the measurement of emotional support relies principally on contact frequency. However, the robustness checks employing closeness as an alternative indicator yielded broadly consistent conclusions; this approach still falls short of fully capturing the rich substantive content of emotional support. Subsequent research could incorporate multidimensional scale instruments to enhance measurement precision. Finally, while this study disaggregates the differentiated effects of community-based home care service utilization on distinct dimensions of intergenerational support behavior, it does not distinguish the heterogeneous impacts of different service types on intergenerational family support, nor does it examine the marginal effects of service utilization intensity. Future research could analyze the differentiated effects of various service types—such as daily living assistance, medical care, and emotional support—and investigate the marginal effects of service frequency and duration, thereby deepening the conclusions.

## 6 Conclusions and recommendations

### 6.1 Research conclusions

As demographic transition and population aging deepen, elder care services have emerged as a critical issue affecting coordinated social development and intergenerational well-being. Focusing on the interaction mechanisms between social elder care and family-based care, this study draws on data from the 2020 Chinese Longitudinal Aging Social Survey (CLASS). It employs the Ordered Probit model, instrumental variable estimation (IV Ordered Probit), and propensity score matching (PSM) to empirically examine the effects of community-based home care service utilization on intergenerational economic support, instrumental support, and emotional support, while also revealing the differential effects of residential arrangements and the urban–rural dual structure. The main conclusions are as follows.

1. Community-based home care service utilization exerts dimensionally differentiated effects on intergenerational family support. With respect to intergenerational economic support, service utilization exhibits a significant enhancement effect, markedly promoting children’s economic support behavior and facilitating more rational household allocation of time and resources. With respect to intergenerational instrumental support and emotional support, service utilization exhibits significant crowding-out effects, partially substituting for the non-economic support functions within the family. These conclusions remain robust after addressing endogeneity, correcting for sample selection bias, and substituting alternative models and measures of intergenerational emotional support.
2. Residential arrangement plays a critical moderating role in the relationship between community-based home care service utilization and intergenerational family support. Co-residence with adult children significantly mitigates the crowding-out effects of service utilization on intergenerational instrumental support and emotional support, indicating that spatial proximity effectively sustains the non-economic support functions within the family by facilitating daily interaction and emotional communication. However, the moderating effect of co-residence on intergenerational economic support is not statistically significant, reflecting the role of digital technologies—such as mobile payment and online transfers—in expanding the channels through which economic support is provided.
3. The effects of community-based home care service utilization on intergenerational family support exhibit pronounced heterogeneity under the urban–rural dual structure. With respect to intergenerational economic support, the promoting effect of service utilization is substantially stronger in urban than in rural areas, attributable to more mature urban labor markets and higher returns to the time released through service substitution. With respect to intergenerational instrumental support and emotional support, the crowding-out effects of service utilization are statistically significant only among urban households, with no significant effect observed among rural households. Plausible explanations include the lower coverage and utilization rates of community-based elder care services in rural areas, where substitution effects have yet to materialize fully, as well as the close-knit kinship networks and traditions of mutual neighborly assistance in rural communities that constitute effective informal support buffers.

### 6.2 Policy recommendations

Building on the research conclusions, the following policy recommendations are proposed.

First, consolidate the foundational role of family-based elder care and guide children in actively fulfilling their intergenerational support responsibilities. The findings indicate that while community-based home care service utilization generates crowding-out effects on instrumental caregiving and emotional interaction, it does not entirely displace family functions, and co-residence with adult children effectively buffers these adverse effects. Accordingly, policy efforts should be directed at three areas. In terms of material incentives, differentiated support measures—such as reductions in social insurance contributions and property tax concessions—should be implemented to alleviate the financial burden of family-based elder care. In terms of time guarantees, the employee family visit leave system should be strengthened, and employers should be encouraged to adopt flexible work arrangements, enabling children to balance occupational demands with filial obligations better and reducing the incidence of care deficits attributable to career pressures. In terms of residential support, coordinated housing and household registration policies should be leveraged to encourage children to reside near or with their parents, thereby enhancing the accessibility and continuity of intergenerational support.

Second, optimize the community-based home care service system to promote “family-community integration” rather than “family-community substitution.” The findings reveal that community-based home care service utilization exerts crowding-out effects on intergenerational instrumental and emotional support; however, this does not suggest that older adults’ use of such services should be curtailed. Rather, it signals the need for policymakers to prioritize preserving intergenerational relationships while encouraging service utilization. Specific measures include: in service content design, incorporating “intergenerational interaction” modules—such as encouraging children to accompany parents in using services and organizing joint family-community activities—to transform service utilization into opportunities for strengthening intergenerational emotional bonds; in service quality improvement, enhancing the emotional communication competencies of service personnel to deliver “person-centered care with warmth,” thereby preventing the weakening of family emotional ties through service provision; and in service model innovation, promoting “caregiver support programs” that, while substituting for a portion of instrumental caregiving, provide children with care skills training and psychological support to reduce caregiving burdens without inducing responsibility dilution.

Third, advance the balanced development of urban and rural elder care services and implement differentiated policy strategies. In response to the low service coverage and utilization rates prevalent in rural areas, fiscal transfer payments should be increased, with priority given to establishing embedded elder care service stations in townships and central villages, thereby expanding the supply of basic, universally accessible services. Concurrently, the advantages of kinship networks inherent in rural, acquaintance-based communities should be fully leveraged to cultivate a hybrid model of “neighborly mutual assistance plus professional services,” rather than simply transplanting urban models. In response to the more pronounced crowding-out effects of service utilization on non-economic support in urban areas, digital technologies—such as WeChat video calls and smart voice assistants—should be harnessed to build integrated “online-offline” interactive platforms that compensate for the caregiving and emotional deficits arising from geographical separation.

## Data Availability

The original contributions presented in the study are included in the article/supplementary material, further inquiries can be directed to the corresponding author.
